# Prediction of microvascular invasion of hepatocellular carcinoma with preoperative diffusion-weighted imaging: A comparison of mean and minimum apparent diffusion coefficient values

**DOI:** 10.1097/MD.0000000000007754

**Published:** 2017-08-18

**Authors:** Jinkun Zhao, Xubin Li, Kun Zhang, Xiaoyu Yin, Xiangfu Meng, Lizhu Han, Xuening Zhang

**Affiliations:** a Department of Radiology, The Second Hospital of Tianjin Medical University; b Department of Radiology, Tianjin Medical University Cancer Institute and Hospital, National Clinical Research Center for Cancer, Tianjin's Clinical Research Center for Cancer, Key Laboratory of Cancer Prevention and Therapy, Huan-hu-xi Road, Hexi District, Tianjin; c Department of Radiology, Linyi Traditional Chinese Medicine Hospital, Shandong, China.

**Keywords:** apparent diffusion coefficient, diffusion-weighted imaging, hepatocellular carcinoma, magnetic resonance imaging, microvascular invasion

## Abstract

The aim of the study was to investigate the value of preoperative diffusion-weighted imaging (DWI) in predicting microvascular invasion (MVI) of hepatocellular carcinoma (HCC), using and comparing mean and minimum apparent diffusion coefficient (ADC) values.

Preoperative MR images of 318 patients with HCC confirmed by surgical pathology were retrospectively analyzed. All patients underwent preoperative DWI on a 1.5 Tesla MRI scanner. The mean and minimum ADC values of the tumors were measured. Interobserver agreements were assessed by the intraclass correlation coefficient (ICC). The ADC values were compared in HCCs between with and without MVI. ROC curves of ADC values were obtained and then compared in distinguishing HCCs with MVI from those without MVI.

There were 211 HCCs with MVI and 107 HCCs without MVI. ICC for the measurements of the mean and minimum ADC values between both observers was 0.88 (95% CI 0.85 – 0.90) and 0.88 (95% CI 0.85 – 0.90), respectively. The mean and minimum ADC values of HCCs with MVI were lower than those of HCCs without MVI (*P* = .00, .00, respectively). With a cut-off value of 0.98 × 10^–3^ mm^2^/s, the minimum ADC (MinADC) showed a sensitivity of 62.56% and a specificity of 65.42% in predicting MVI, whereas the mean ADC provided a sensitivity of 79.15% and a specificity of 50.47% with a cut-off value of 1.19 × 10^–3^ mm^2^/s. No significant difference existed between MinADC and mean ADC for their diagnostic performances in the prediction of MVI (*P* = .48).

DWI could preoperatively provide quantitative parameters for predicting MVI of HCC.

## 1 Introduction

As the most common primary hepatic malignancy, the incidence of hepatocellular carcinoma (HCC) is increasing, especially in Western nations.^[1]^ The high recurrence rate is a major problematic issue for hepatic resection with regard to achieving cure and long-term survival, because the tumor recurrence rate exceeds 60% at 5 years even in patients with small tumors.^[2]^ It has been reported that microvascular invasion (MVI) is the most important independent risk factor affecting recurrence and survival in patients with HCCs after curative resection.^[3]^ After liver transplantation, MVI positivity shortens the disease-free survival at 3 years (relative risk, 3.41) and overall survival at 3 and 5 years (relative risk, 2.41 and 2.29, respectively) and, also after hepatic resection, MVI positivity impacts disease-free survival at 3 and 5 years (relative risk,1.82 and 1.51, respectively).^[4]^ These research results suggest that it is important to predict the presence of MVI before hepatic resection for help in determining treatment strategies. To eradicate MVI, anatomical hepatic resection ^[5]^ or partial hepatic resection with a wide tumor margin^[6]^ is recommended.

In contrast to macrovascular invasion, conventional imaging modalities have been ineffective for the preoperative detection of MVI because MVI is a microscopic parameter.^[7]^ Moreover, the detection of MVI by using preoperative biopsy has proven unreliable because of the intratumoral heterogeneity that causes the sampling error.^[4]^ MVI is mainly diagnosed only after surgical treatments by means of histopathologic evaluation.

Diffusion-weighted imaging (DWI) with apparent diffusion coefficient (ADC) analysis has been widely used for tumor characterization in clinical practice.^[8]^ It has been reported that ADC is a useful imaging biomarker in diagnosis and predicting tumor grade and treatment response of HCC.^[9–11]^ In addition, some studies show that DWI has also been useful in predicting MVI for HCC.^[12,13]^ However, these studies selected different *b* values for DWI. The research results need to be further verified to be reproducible and reliable. Moreover, these studies had limited numbers of cases and just focused on the mean ADC value, not including the minimum ADC (MinADC) value. Thus, the objective of our study based on a relative larger number of cases was to characterize the role of tumor ADC (minimum and mean ADC) values in preoperative detection of MVI in patients with HCCs.

## 2 Methods

### 2.1 Study population

This retrospective study was approved by the Institutional Review Board of Tianjin Medical University Cancer Institute and Hospital, and written informed consent was waived from all patients. A total of 378 patients with histologically proven HCC underwent preoperative upper abdominal MRI including DWI at our institution between January 2011 and January 2015. Among the patients, 42 patients who received transarterial chemoembolization or radiofrequency ablation before MRI and 18 patients with combined hepatocellular-cholangiocarcinoma were excluded. Finally, 318 patients were enrolled in the present study. The study group consisted of 258 men and 60 women patients with mean age of 59 years and age range of 19 to 87 years. MR images, surgical records, and pathological reports were available in all cases. The patients’ demographic and clinical characteristics were recorded, including age, gender, background liver parenchymal disease, history of hepatitis, and the largest diameter of the tumor. All patients were performed hepatic resection surgery in 1 week after MR examination. None of the patients who were enrolled in the present study had previously undergone any treatment before examination.

### 2.2 MR imaging protocol

All participants were imaged using a 1.5 Tesla GE MRI scanner (Signa Excite HD, GE Healthcare, Milwaukee, WI) equipped with an 8-channel phased-array coil. Initially, breath-hold axial T1-weighted MR (T1W) images with gradient-echo sequences (fast spoiled gradient-echo; repetition time [TR], 140–170 ms; echo time [TE], 1.8 ms and 4.2 ms; flip angle, 90) and respiratory-triggered fat-saturated fast spin-echo T2-weighted MR images (T2W; TR/TE = 7059/80 ms) were obtained with slice thickness, 7 mm; space gap, 1 mm; field of view (FOV), 40 cm × 34 cm; matrix, 128 × 128 or 320 × 160. Before contrast agent injection, a respiratory-triggered DWI was performed using the following parameters: sequence, single-shot spin-echo echo-planar with a parallel imaging technique (factor = 2); fat-saturated technique; scan direction, axial; *b* value, 0 s/mm^2^ and 800 s/mm^2^; directions of diffusion gradients, 3 orthogonal directions; TR/TE 4225/61.4 ms; matrix, 128 × 128; slice thickness, 8 mm; space gap, 1 mm; slices 24; field of view, 38–40 cm × 38–40 cm; excitation number, 4 and acquisition time, 93 s. Subsequently, an axial breath-hold T1-weighted 3D fat suppressed spoiled gradient-echo (GRE) sequence (Liver Acquisition with Volume Acceleration, LAVA) was used for dynamic contrast-enhanced imaging with the following parameters: TR/TE 4.8/1.7 ms; flip angle, 15°; FOV, 315 × 360; matrix, 256 × 256; section thickness, 4 mm. The contrast agent was applied in terms of a bolus injection of 0.1 mmol/kg body weight of gadopentetate dimeglumine (Magnevist, Bayer Schering, Berlin, Germany) and images were acquired during the hepatic arterial (20 s delay), portal venous (60 s delay), and equilibrium phases (180 s delay). The scan delay time was prolonged 5 to 6 minutes after injection if needed.

### 2.3 Image analysis

First, images were retrospectively reviewed by 2 radiologists (Li and Zhang, with 6 and 3 years of experience in interpretation of abdominal MR images, respectively), independently. Then, statistical analysis was performed to evaluate interobserver variability. Both radiologists agreed that if a non-statistically significant result was obtained, the final image analysis would be performed by the 2 abdominal radiologists with consensus. Both observers were blinded to the histological information, but were aware of the patients’ clinical information.

Diffusion-weighted images were viewed in conjunction with conventional and contrast-enhanced MR images for anatomical correlation of the lesion. ADC maps were generated from DWI with commercial diffusion-analysis software using a monoexponential fit (Functool version 4.6, GE Healthcare, Milwaukee, WI). ADC values of the regions of interest (ROIs) were measured on ADC maps at the same workstation by both observers.

A free-hand ROI was manually and carefully drawn on each slice of tumor ADC maps, which included the solid portion of a tumor as far as possible, avoiding the cystic, necrotic, or hemorrhagic regions in the tumor. To ensure the accurate placement of the ROIs on ADC maps, the ROIs were obtained by transferring the ROIs on DWI to the ADC maps at the corresponding slice positions. The ROIs were drawn on DWI using the fat-saturated T2WI images as a reference. The mean ROI area was 1324 mm^2^ (range, 28–8523 mm^2^). The number of the slice depended on the size of the tumor, ranging from 2 to 19. The ADC value of each ROI was recorded. For each observer, the average of all the ADC values derived from individual-slice ROI of a tumor was regarded as the mean ADC value of the tumor and the lowest ADC value among the ADC values derived from individual-slice ROI was defined as the MinADC value of the tumor, respectively. Then, statistical analysis was performed to evaluate the interobserver variabilities of the mean ADC and the MinADC values, respectively. If a nonstatistically significant result of interobserver variability assessment is obtained, the final assessment of the mean and minimum ADC values of the tumors will be performed by the 2 abdominal radiologists with consensus in a few days after interobserver variability assessment.

### 2.4 Statistical analysis

Statistical analysis was performed by using SPSS v. 17.0 (Chicago, IL) and MedCalc v. 16.2.1 (Mariakerke, Belgium). Interobserver agreement was assessed by the intraclass correlation coefficient (ICC) for the ADC value measurements. Additionally, for the mean and minimum ADC values, a Bland–Altman plot was constructed between both observers, respectively. ICC values were categorized into 5 categories: 0.0 to 0.20 as poor; 0.21 to 0.40 as fair; 0.41 to 0.60 as moderate; 0.61 to 0.80 as good; 0.81 to 1.00 as excellent. The mean and minimum ADC values were respectively compared in HCCs between with and without MVI with 1-way analysis of variance (ANOVA). Receiver operating characteristic (ROC) curves of mean and minimum ADC values were also performed to calculate the best cutoff value with the sensitivity and specificity to distinguish HCCs with MVI from those without MVI and then compared. *P* < .05 was considered to indicate a statistically significant difference.

## 3 Results

Based on the surgical and pathological records, 318 HCCs were enrolled in the present study. Among the tumors, there were 211 HCCs with MVI and 107 HCCs without MVI. The largest diameter of the tumors ranged from 1.5 cm to 21.0 cm with a mean diameter of 5.7 cm. With regard to age, gender, background liver parenchymal disease (including cirrhosis and chronic hepatitis), and the largest diameter of the tumor, there were no statistically significant differences between the MVI-positive and MVI-negative groups (Table 1).

**Table 1:**
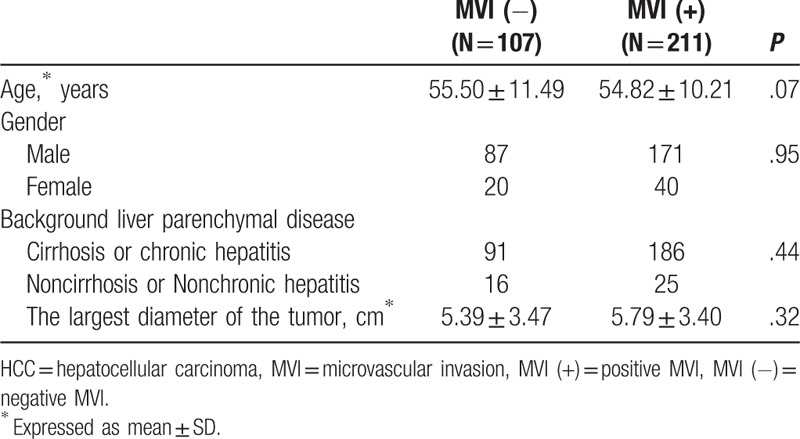
Summary of patients’ characteristics for the histopathological MVI of HCCs.

**Figure 1:**
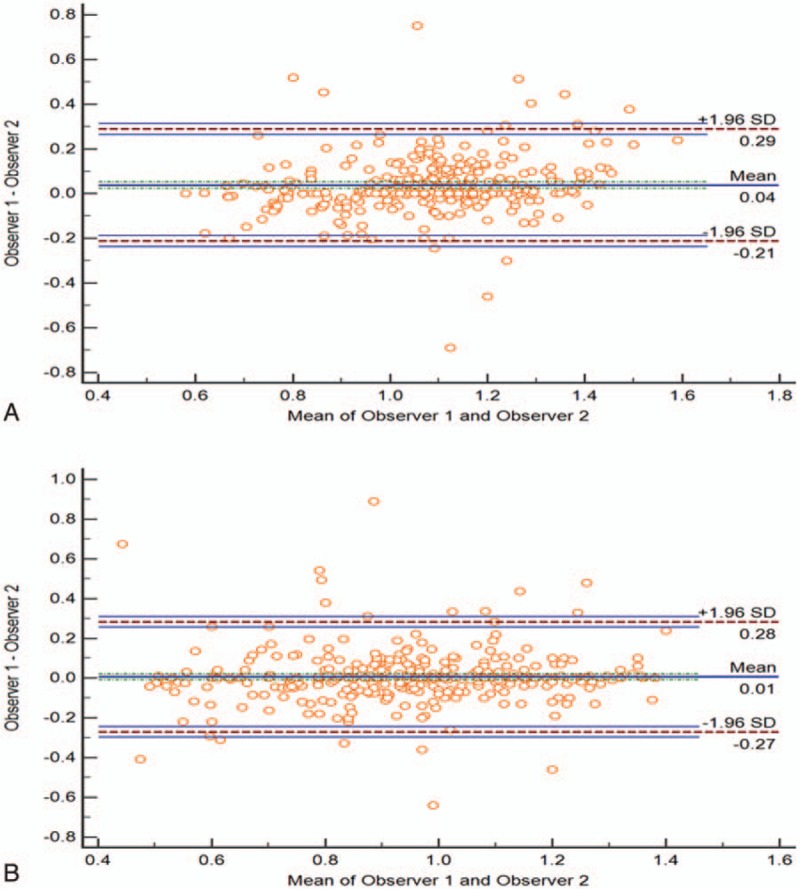
Bland–Altman plots show the measurements of the mean and MinADC values, comparing the interobserver agreement. Plot A shows the results of the mean ADC value between both observers and plot B shows the results of the MinADC value between both observers. The solid line indicates mean difference and the dash line indicates 95% limits of agreement. ADC = apparent diffusion coefficient, MinADC = minimum ADC.

There were excellent interobserver agreements for the measurements of the mean and minimum ADC values, respectively. ICC between both observers was 0.88 (95% CI 0.85 – 0.90) for the mean ADC value, and 0.88 (95% CI 0.85 – 0.90) for the minimum ADC value. Bland–Altman plots with respect to the mean and minimum ADC values show that mean difference between both observers is 0.04 × 10^–3^ mm^2^/s (limits of agreement –0.21 to 0.29) for the mean ADC value and 0.01 × 10^–3^ mm^2^/s (limits of agreement –0.27 to 0.28) for the minimum ADC value (Fig. 1).

**Figure 2:**
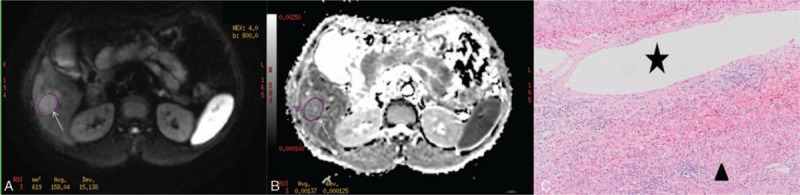
A moderate-differentiated hepatocellular carcinoma without microvascular invasion in a 56-year-old man. (A) DWI obtained with *b*-value of 800 s/mm^2^ shows a lesion (arrow) with higher signal intensity at segment VI of the liver. (B) On the corresponding ADC map, the ADC value of the lesion (arrow) was 1.37 × 10^−3^ mm^2^/s. (C) Microvascular invasion (star) was not detected around the tumor (arrowhead) by a microscopic examination (hematoxylin-eosin stain original magnification ×200). ADC = apparent diffusion coefficient.

**Figure 3:**
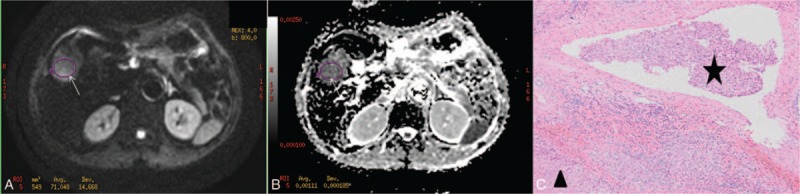
A moderate-differentiated hepatocellular carcinoma with microvascular invasion in a 73-year-old man. (A) DWI obtained with *b*-value of 800 s/mm^2^ shows a lesion (arrow) with higher signal intensity at segment V of the liver. (B) On the corresponding ADC map, the ADC value of the lesion (arrow) was 1.11 × 10^−3^ mm^2^/s. (C) Microvascular invasion (star) was observed around the tumor (arrowhead) by a microscopic examination (hematoxylin-eosin stain original magnification ×200). ADC = apparent diffusion coefficient, DWI = diffusion-weighted imaging.

**Figure 4:**
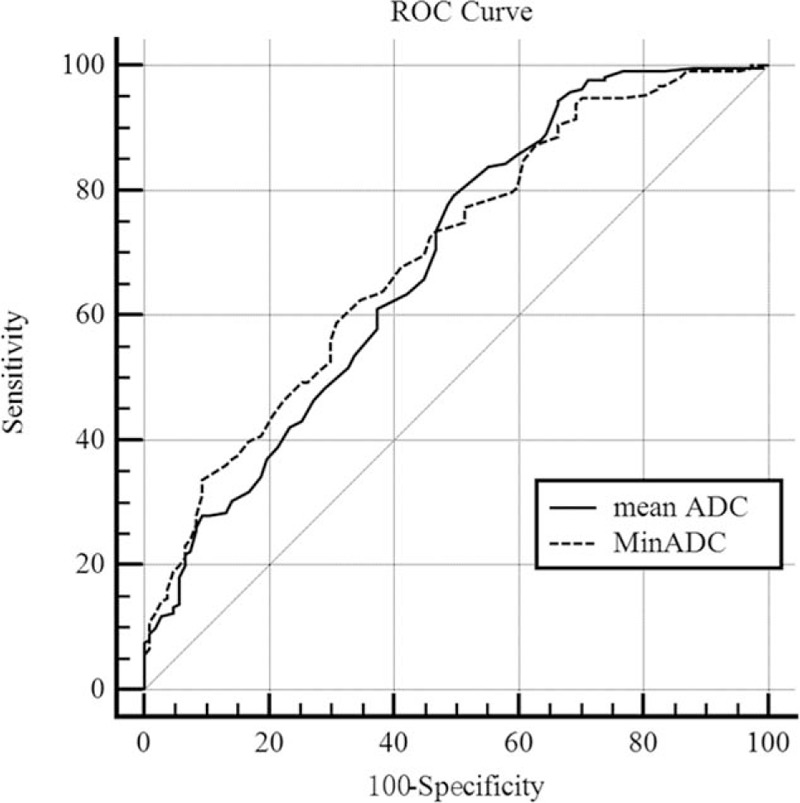
ROC analysis demonstrates that no significant difference exists between MinADC value with an AUC of 0.70 (dash line) and mean ADC value with an AUC of 0.69 (solid line) in distinguishing HCCs with MVI from those without MVI. ADC = apparent diffusion coefficient, AUC=area under the curve, HCC = hepatocellular carcinoma, MinADC = minimum ADC, MVI = microvascular invasion, ROC = receiver operating characteristics curve.

The mean and minimum ADC values of HCCs in male patients were respectively 1.11 × 10^–3^ mm^2^/s and 0.96 × 10^–3^ mm^2^/s, whereas those in female patients were respectively 1.11 × 10^–3^ mm^2^/s and 0.98 × 10^–3^ mm^2^/s. No significant differences existed between male and female patients in both mean and minimum ADC values, (*P* = .91, .59, respectively). The mean ADC values of HCCs with and without MVI were respectively 1.07 × 10^–3^ mm^2^/s and 1.19 × 10^–3^ mm^2^/s, whereas their MinADC values were 0.92 × 10^–3^ mm^2^/s and 1.06 × 10^–3^ mm^2^/s, respectively. The mean and minimum ADC values of HCCs with MVI were lower than those of HCCs without MVI (*P* = .00, .00, respectively) (Figs. 2 and 3, Table 2). With a cut-off value of 0.98 × 10^–3^ mm^2^/s, the minimum ADC (MinADC) showed a sensitivity of 62.56%, a specificity of 65.42%, a positive likelihood ratio (+LR) of 1.81, and a negative likelihood ratio (–LR) of 0.57 in predicting MVI, whereas the mean ADC provided a sensitivity of 79.15% and a specificity of 50.47%, a +LR of 1.60 and a –LR of 0.41 with a cut-off value of 1.19 × 10^–3^ mm^2^/s. No significant difference existed between MinADC and mean ADC for their diagnostic performances in the prediction of MVI (*P* = .48, Fig. 4).

**Table 2:**
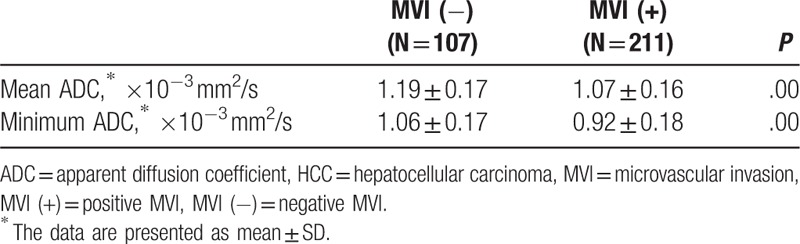
Comparisons of minimum and mean ADC values between HCCs with and without MVI, respectively.

## 4 Discussion

MVI is the most important independent risk factor affecting recurrence and survival in HCC patients after curative resection.^[3]^ Preoperative prediction of MVI for HCC would be helpful to make a surgical planning, choose an optimal therapeutic method and predict the prognosis. Previous studies which were based on limited cases focused on the mean ADC value and showed that the ADC value derived from DWI was a useful imaging biomarker in preoperatively predicting MVI of HCC.^[12,13]^ To verify the results’ reproducibility and reliability, our present study compared the roles of the mean and minimum ADC values in the prediction of MVI in HCCs based on a relative larger number of cases. Our results demonstrated that the mean and minimum ADC values of HCCs with MVI were lower than those of HCCs without MVI and no significant difference existed between MinADC and mean ADC for their diagnostic performances in predicting MVI. These results suggest DWI could provide quantitative parameters in the preoperative prediction of MVI in HCC.

DWI has been extensively used as a cancer-imaging tool in clinical practice. It has been reported that the lower ADC value can preoperatively predict MVI in HCC,^[12,13]^ which is consistent with our research results. However, its specificity in our study is much lower than those reported in previous studies (50.47% vs 78.6%, 72.2%, respectively). The discriminations may be explained by the different choices of the *b* values when DWI was performed and the method of measuring ADC values. In our study, common *b* values of abdominal DWI (*b* = 0, 800 s/mm^2^) were used and ROIs were located on the solid portions of a tumor to avoid the cystic or necrotic portion on each ADC map image of the tumor, which are different from those used in previous studies (*b* value: 0 and 500 s/mm^2^ or 50, 400, and 800 s/mm^2^; ROI: including the entire lesion).^[12,13]^ Therefore, an internationally standardized ADC measuring method and *b* value choice of abdominal DWI should be adopted in the future studies. Although this, in our present study, compared to HCCs without MVI, HCCs with MVI still showed lower mean ADC values, which demonstrated a statistically significant difference. The result suggests that the mean ADC value could be a useful predictor of MVI during the preoperative evaluation of HCCs.

Heterogeneity, an important biological feature of tumors could be reflected by heterogeneous ADC values. Theoretically, the MinADC value of a tumor corresponds with the highest tumor cellularity, which is also the most actively proliferative area.^[14]^ Studies have suggested that the MinADC may be an effective parameter for differentiation between malignant and benign breast lesions and the prediction of tumor grading.^[14–17]^ On the basis of these studies, we hypothesized that the MinADC value might be another useful quantitative parameter in the preoperative prediction of MVI in HCC. Our present study results showed that the MinADC values of HCCs with MVI were lower than those of HCCs without MVI, which demonstrated a statistically significant difference. This agrees with our hypothesis. Moreover, its specificity was much higher than the mean ADC value. However, no significant difference existed between the MinADC value and the mean ADC value in the preoperative prediction of MVI. The reason could be that the MinADC value had a relative lower sensitivity than the mean ADC value.

Our research results demonstrated that compared to HCCs without MVI, HCCs with MVI showed lower both mean and minimum ADC values. The reasons are still unclear. The following possible mechanisms may be suggested, although they are not confirmed by histopathological analysis. First, HCCs with MVI may have higher cellularity with restricted diffusion than HCCs without MVI, although it is unknown whether the higher cellularity of HCCs with MVI is the cause of or result of MVI.^[12,13]^ Second, DWI and ADC values provide information related to the tissue cellularity and integrity of cellular membranes, as well as microcapillary perfusion by reflecting the molecular diffusion of water and perfusion. HCCs with MVI may have decreased perfusion, which causes the lower mean and minimum ADC values. Intravoxel incoherent motion imaging (IVIM) can provide the information about the alteration of perfusion in liver.^[18,19]^ Therefore, to verify the hypothesis, further studies with IVIM may be recommended.

There are several limitations in our study. First, because of its retrospective nature, the possibility of a selection bias cannot be excluded. Second, the ADC measuring method and abdominal DWI protocol used in our study could cause the discrepancy between our and previous studies to some extent. Their standardizations need to be further investigated. Third, for the prediction of MVI, we did not compare the relative accuracy of ADC values and other imaging findings that have been reported in previous studies^[20,21]^ because our main goal was to characterize the value of DWI in the prediction of MVI. Future studies should compare DWI findings, conventional imaging findings, and a combination of the two. Finally, we performed DWI using only 2 *b* values of 0 and 800 s/mm^2^ in our study. Theoretically, ADC measurement with DWI obtained with multiple *b*-values might reduce the measurement error, thus potentially improving reproducibility. Intravoxel incoherent motion DW-MR imaging applying multiple *b* values might be a promising tool to improve data reproducibility and detect MVI of HCC.

In conclusion, HCCs with MVI had lower both mean and minimum ADC values than HCCs without MVI. No significant difference existed between MinADC and mean ADC for their diagnostic performances in the prediction of MVI. MinADC and mean ADC values derived from DWI could be used as quantitative predictors of MVI during the preoperative evaluation of HCCs.
